# Risk of serous retinal detachment in patients with end-stage renal disease on dialysis

**DOI:** 10.1371/journal.pone.0180133

**Published:** 2017-06-28

**Authors:** Yuh-Shin Chang, Shih-Feng Weng, Chun Chang, Jhi-Joung Wang, Hong-I Chen, Shun-Yao Ko, I-Te Tu, Chih-Chiang Chien, Jian-Jhong Wang, Ching-Min Wang, Ren-Long Jan

**Affiliations:** 1 Department of Ophthalmology, Chi Mei Medical Center, Tainan, Taiwan; 2 Graduate Institute of Medical Science, College of Health Science, Chang Jung Christian University, Tainan, Taiwan; 3 Department of Healthcare Administration and Medical Informatics, Kaohsiung Medical University, Kaohsiung, Taiwan; 4 Department of Education, University of Taipei, Taipei, Taiwan; 5 Department of Medical Research, Chi Mei Medical Center, Tainan, Taiwan; 6 Department of Anesthesiology, Chi Mei Medical Center, Tainan, Taiwan; 7 Department of Internal Medicine, Chi Mei Medical Center, Liouying, Tainan, Taiwan; 8 Department of Internal Medicine, Chi Mei Medical Center, Tainan, Taiwan; 9 Graduate Institute of Clinical Medicine, National Cheng Kung University, Tainan, Taiwan; 10 Department of Pediatrics, Chi Mei Medical Center, Liouying, Tainan, Taiwan; Hospital Universitario de la Princesa, SPAIN

## Abstract

The aim of this retrospective, nationwide, matched cohort study was to investigate the association of serous retinal detachment with having end-stage renal disease (ESRD) while on dialysis. The cohort study included 94,024 patients with ESRD on dialysis registered between January 2000 to December 2009 in the Taiwan National Health Insurance Research Database. An age- and sex-matched control group comprised 94,024 patients selected from the Taiwan Longitudinal Health Insurance Database 2000. Information for each patient was collected from the index date until December 2011. Twenty-seven ESRD patients and 11 controls developed serous retinal detachment (P < 0.001) during follow-up, demonstrating a significantly increased risk of serous retinal detachment in patients with ESRD on dialysis compared with controls (incidence rate ratio = 3.39, 95% confidence interval [CI] = 1.68–6.83). After adjustment for potential confounders, patients were 3.86 times more likely to develop serous retinal detachment than the full cohort (adjusted HR = 3.86, 95% CI = 1.15–12.96). In conclusion, patients with ESRD on dialysis demonstrate an increased risk of serous retinal detachment. Interdisciplinary collaboration between nephrologists and ophthalmologists is important to deal with serous retinal detachment in patients with ESRD on dialysis and prevent impairments of visual acuity.

## Introduction

End-stage renal disease (ESRD), the most severe form of chronic kidney disease, is known as a worldwide public health problem. ESRD often requires dialysis or kidney transplantation because of the poor prognosis owing to morbidity and mortality [1–3]. The prevalence and incidence of patients with ESRD on dialysis have continued to increase in both Western and Asian populations [4,5]. Taiwan has the highest incidence and prevalence of patients with ESRD on dialysis [6–8]. Furthermore, the association between ESRD and many retinal diseases, such as retinal artery occlusion, retinal vein occlusion, or age-related macular degeneration, has been demonstrated by several reports recently [9–11].

Serous retinal detachment occurs when the neurosensory retina separates from the pigment epithelium leading to the visual impairment. Serous retinal detachment is usually accompanied by retinal pigment epithelial detachments where the retinal pigment epithelium separates from Bruch’s membrane of the choroid and results in acute vision disturbance [12,13]. Furthermore, the initial retinal pigment epithelial detachment may be the cause of the development of a subsequent serous retinal detachment. Although the exact mechanisms of sub-retinal exudation is not well understood, they are thought to include increased choroidal vascular permeability and perfusion, destabilization of the retinal epithelium, and increased choroidal interstitial fluid with further extension into the sub-retinal space [12,13]. The causes of serous retinal detachment are multi-factorial and include systemic inflammatory diseases [14,15], infectious diseases [16–18], collagen vascular diseases [13], disseminated intravascular coagulopathy [13], and malignant disease [19–21].

The occurrence of serous retinal detachment has been described in patients receiving haemodialysis in several previous reports [22–24]. However, the underlying mechanism of serous retinal detachment is not well understood and controversial. Although Gass has suggested that the uraemic state is more important than haemodialysis in the development of serous retinal detachment [22], Troiano et al. hypothesized that haemodialysis is the more important cause of the phenomenon [23]. In addition, previous studies that discussed the association between ESRD treated with dialysis and serous retinal detachment were limited by the small number of patients or the absence of comparative control data [12,13,22–24]. By using a nationwide population-based dataset, we designed a cohort study to investigate the risk of serous retinal detachment in patients with ESRD on dialysis in Taiwan.

## Methods

### Database

In Taiwan, a single-payer National Health Insurance (NHI) scheme was launched on March 1, 1995, which provides extensive medical care coverage for all residents. As of 2007, about 22.60 million individuals (>98%) of the total Taiwanese population of 22.96 million were enrolled in this program. Our cohort study data were obtained from the Taiwan National Health Insurance Research Database (NHIRD). The NHIRD supplies enciphered patient identification numbers, and information regarding patient date of birth, sex, and admission and discharge dates. The International Classification of Diseases, Ninth Revision, Clinical Modification (ICD-9-CM) diagnoses and procedure codes, prescriptions details, and costs covered and paid by the NHI scheme are also included in the NHIRD. Ethical approval and informed consent were waived by the Institutional Review Board of Chi-Mei Medical Center because a public database was used for the analysis. Because the analysis of datasets in a database does not use identifiable personal information, the requirement of informed consent was waived.

### Study design

This retrospective, nationwide, matched cohort study involved two groups of participants: a new-onset ESRD on dialysis patient group and a matched non-ESRD on dialysis control group.

### Study participants

In our study, we included 94,024 patients with ESRD who started receiving dialysis treatment after 31 December 2000 and who had received a catastrophic illness certificate with the code number 585 between 1 January 2000 and 31 December 2009. Patients with unknown sex or missing data were excluded. Patients diagnosed as having serous retinal detachment (ICD-9-CM codes 361.2, 361.42, or 361.43) prior to ESRD were also excluded.

For each patient with ESRD on dialysis, one patient without ESRD on dialysis was randomly selected from the longitudinal Health Insurance Database 2000 (LHID2000). LHID2000 is a data subset of the NHIRD that contains all claim data for one million beneficiaries (4.34% of the total population) randomly selected in 2000. There were no significant differences in age, sex, or health care costs between the sample group and all national health insurance enrolees between the NHIRD and LHID 2000. The 94,024 controls were matched by age, sex, and index date. The index date for the patients with ESRD on dialysis was the date of their first dialysis, and the index date for the controls was created by matching the date with the index date of the patient with ESRD on dialysis. Controls diagnosed with serous retinal detachment before the index date were also excluded. Each patient was followed up to determine the incidence of serous retinal detachment until the end of 2011 or censored because of death.

To distinguish all patients who had developed serous retinal detachment (ICD-9-CM codes 361.2, 362.42, or 362.43), we tracked every patient from his or her index outpatient visit or hospitalization until December 2011. Demographic data (e.g. age, sex, follow-up period, and dialysis type) were recorded. Furthermore, we collected information regarding comorbidities including diabetes mellitus (ICD-9-CM code 250), chorioretinal inflammation (ICD-9-CM code 363.0, 363.1, 363.2), and malignant neoplasm of eye (ICD-9-CM code 190), because these conditions are critical factors that increase the risk of serous retinal detachment. In the study, the inclusion criteria for the above comorbidities were documentation of the condition at least once in the inpatient setting or ≥3 times in the ambulatory setting within 1 year before the initial ESRD on dialysis medical service date.

### Statistical analysis

SAS 9.4 for Windows (SAS Institute, Inc., Cary, NC, USA) was used in this study. We compared the demographic characteristics and comorbid disorders between the ESRD on dialysis and control groups with Pearson chi-square test. The incidence rate was calculated as the number of cases of serous retinal detachment identified during follow-up divided by the total person-years (PY) for each group by age, sex, and select comorbidities. The incidence rate ratio (IRR), which demonstrates the comparison in the risk of developing serous retinal detachment between the ESRD on dialysis and control groups, was calculated using Poisson regression analysis. The conditional Cox proportional hazard regression analysis (in SAS, phreg with strata) was performed to calculate the adjusted hazard ratio (HR) for developing serous retinal detachment in the two groups. In addition, to avoid significant bias due to small events in our study, the conditional proportion hazard regression adjusted by the Firth method was used to estimate the risk. Cumulative incidence rates for serous retinal detachment of patients with ESRD on dialysis were evaluated by Kaplan–Meier analysis, and the difference in cumulative-incidence rate curves was analysed using the log-rank test. Additionally, we subdivided the patients into three age subgroups for further analysis: <50 years, 50–64 years, and ≥65 years. Additionally, we subdivided the patients into two dialysis subgroups—haemodialysis (HD) and peritoneal dialysis (PD)—to evaluate whether patients with ESRD on different dialysis modalities would have different risks of serous retinal detachment. Data are presented as mean±standard deviation (SD), and 95% confidence intervals (CIs) are provided when applicable. Statistical significance was defined as P<0.05. These statistical assessments were performed in consultation with a statistical expert.

## Results

### Demographic data

Between 2000 and 2009, 94,024 patients with ESRD on dialysis and 94,024 controls were recruited after excluding ineligible subjects. Table 1 provides the demographic characteristics and comorbid disorders of the patients with ESRD on dialysis and the age- and sex-matched controls. The mean age of all participants was 62.23±14.64 years. Patients with ESRD on dialysis exhibited a significantly higher prevalence of previously reported comorbidities such as diabetes, and chorioretinal inflammation, than the controls. The mean follow-up periods for the patients with ESRD on dialysis and control patients were 4.71±3.26 and 6.50±2.95 years, respectively. We classified the patients with ESRD on dialysis into HD and PD groups based on the different treatment modalities, finding that of the 94,024 patients with ESRD on dialysis, 83,764 (89.09%) received HD and 10,260 (10.91%) received PD (Table 1).

**Table 1:** Demographic characteristics and comorbid disorder comparisons between the ESRD on dialysis and control groups.

	ESRD on dialysis (N = 94,024)	Controls (N = 94,024)	
	n (%)	n (%)	*P*-value
**Age on the index date (mean±SD)**	62.23±14.64	62.23±14.64	1.0000
**Age on the index date**			
**<50**	18,235 (19.39)	18,235 (19.39)	1.0000
**50–64**	30,051 (31.96)	30,051 (31.96)	
**≥65**	45,738 (48.65)	45,738 (48.65)	
**Sex**			
**Male**	47,441 (50.46)	47,441 (50.46)	1.0000
**Female**	46,583 (49.54)	46,583 (49.54)	
**Baseline comorbidity**			
**DM**	49,617 (52.77)	10,337 (10.99)	<0.001
**Chorioretinal inflammation**	13 (0.01)	4 (<0.001)	0.0290
**Malignant neoplasm of eye**	10 (0.01)	6 (0.01)	0.3173
**Follow-up (years) (mean±SD)**	4.71±3.26	6.50±2.95	< 0.001
**Dialysis type**			
**HD**	83,764 (89.09)		
**PD**	10,260 (10.91)		

Note: Demographic characteristics and comorbid disorders were compared between the ESRD on dialysis and control groups by Pearson’s chi-squared tests. Abbreviations: DM, diabetes mellitus; ESRD: end-stage renal disease; HD, haemodialysis; PD, peritoneal dialysis; SD, standard deviation.

### Incidence rates of serous retinal detachment

During the follow-up period, 38 (38/188,048 [0.02%]) patients developed serous retinal detachment. A significantly higher proportion of patients with ESRD on dialysis (27/94,024 [0.03%]) than control patients (11/94,024 [0.01%]) developed serous retinal detachment (Table 2). In addition, there was a significant difference in the incidence of serous retinal detachment between the groups (patients with ESRD on dialysis = 0.61/10000 PY; control = 0.18/10000 PY), and the IRR between the ESRD on dialysis and control groups was statistically significant (3.39, 95% CI = 1.68–6.83, P<0.001; Table 2).

**Table 2:** Risk of serous retinal detachment in the ESRD on dialysis and control groups.

Characteristics	ESRD on dialysis				Controls				IRR (95% CI)	*P*-value
	N	Serous RD	PY	Rate^a^	N	Serous RD	PY	Rate^a^		
**All**	94,024	27	443,149.74	0.61	94024	11	611,366.99	0.18	3.39 (1.68–6.83)	<0.001
**Age (years)**										
**<50**	18,235	13	114,782.7	0.11	18235	0	132,011	-	-	
**50–64**	30,051	9	151,229.8	0.60	30051	6	200,037.26	0.30	1.98 (0.71–5.57)	0.1936
**≥65**	45,738	5	177,137.23	0.28	45738	5	279,318.74	0.18	1.58 (0.46–5.46)	0.4715
**Sex**										
**Male**	46,583	15	213,488.9	0.70	46583	7	296,882.82	0.24	2.98 (1.21–7.31)	0.017
**Female**	47,441	12	229,660.83	0.52	47441	4	314,484.17	0.13	4.11 (1.32–12.74)	0.014
**Comorbidity**										
**DM**	49,617	12	20,4080.5	0.06	10337	0	58,740.33	-	-	
**Chorioretinal inflammation**	13	0	80.59	0	4	0	15.64	0	-	
**Malignant neoplasm of eye**	10	0	32.72	0	6	0	30.98	0	-	

Note: A Poisson regression analysis was performed to calculate the incidence rate ratio. Abbreviations: CI: confidence interval; DM, diabetes mellitus; ESRD, end-stage renal disease; IRR, incidence rate ratio; PY, person-years; RD, retinal detachment.

^a^Rate: per 10,000 person-years.

After the two groups were divided by age, we found that patients with ESRD on dialysis aged 50 to 64 years had the highest incidence rate (0.60/10000 PY), followed by patients ≥65 years old, and patients <50 years old. (Table 2)

Male patients with ESRD on dialysis had an incidence of serous retinal detachment of 0.70/10000 PY, whereas that male control patients had an incidence of only 0.24/10000 PY, leading to a significant IRR (IRR = 2.98, 95% CI = 1.21–7.31, P = 0.017). For female patients, a significant difference was also noted between patients with ESRD on dialysis and their controls (IRR = 4.11, 95% CI = 1.32–12.74, P = 0.014; Table 2).

Regarding comorbidities, serous retinal detachment only occurred in the subgroup of patients with diabetes within the ESRD on dialysis group. In that case, incidence rates were as high as 0.06/10000 PY. However, the IRR for serous retinal detachment associated with diabetes mellitus could not be determined because no serous retinal detachment was observed in patients with diabetes mellitus in the control group. There was no incidence of serous retinal detachment among patients with chorioretinal inflammation, nor with malignant neoplasm of eye, in the ESRD on dialysis group. We were unable to evaluate whether chorioretinal inflammation or malignant neoplasm of eye in patients with ESRD increases the risk of serous retinal detachment, because the incidence of serous retinal detachment among such patients in both groups was essentially zero (Table 2).

Table 3 provides the crude and adjusted HRs for serous retinal detachment, by cohort, during the follow-up period. After adjusting for select comorbid conditions, ESRD on dialysis remained an independent risk factor for serous retinal detachment (adjusted HR = 3.86, 95% CI = 1.15–12.96). Both HD and PD were independent risk factors for the development of serous retinal detachment after adjusting for other confounding factors in the total cohort (adjusted HR [95% CI] = 6.47 [2.40–17.44] for PD and 2.95 [1.35–6.44] for HD). The risk of serous retinal detachment in patients who underwent PD was higher than patients who underwent HD. No comorbidities were significant risk factors for serous retinal detachment in either group after adjusting for select comorbid conditions. Finally, the lack of occurrences of serous retinal detachment among patients with chorioretinal inflammation or malignant neoplasm of eye prevented us from evaluating whether such conditions were significant risk factors in the total cohort, after adjusting for other confounding factors.

**Table 3:** Crude and adjusted hazard ratios of conditional Cox proportional hazard regressions and 95% confidence intervals with the Firth method for serous retinal detachment during the follow-up period in the study cohort.

Cohort	Crude Hazard Ratio (95% CI)	Adjusted Hazard Ratio (95% CI)
**ESRD on dialysis**		
**No**	1.00	1.00
**Yes**	2.88* (1.31–6.36)	3.86* (1.15–2.96)
**PD**	6.21* (2.32–16.57)	6.47* (2.40–17.44)
**HD**	2.70* (1.30–5.62)	2.95* (1.35–6.44)
**Comorbidity**		
**DM**		
**No**	1.00	1.00
**Yes**	2.09 (0.73–5.96)	0.54 (0.11–2.69)
**Chorioretinal inflammation**		
**No**	1.00	1.00
**Yes**	–	–
**Malignant neoplasm of eye**		
**No**	1.00	1.00
**Yes**	–	–

Note: The adjusted hazard ratio for developing serous retinal detachment was calculated using a conditional Cox proportional hazard regression analysis with the Firth method. Because no serous retinal detachment event occurred in the patients with either chorioretinal inflammation or malignant neoplasm of eye, the hazard ratios were not calculated in the model. Abbreviations: CI: confidence interval; DM, diabetes mellitus; ESRD: end-stage renal disease; HD, haemodialysis; PD, peritoneal dialysis.

*P-value < .05.

**Fig 1:**
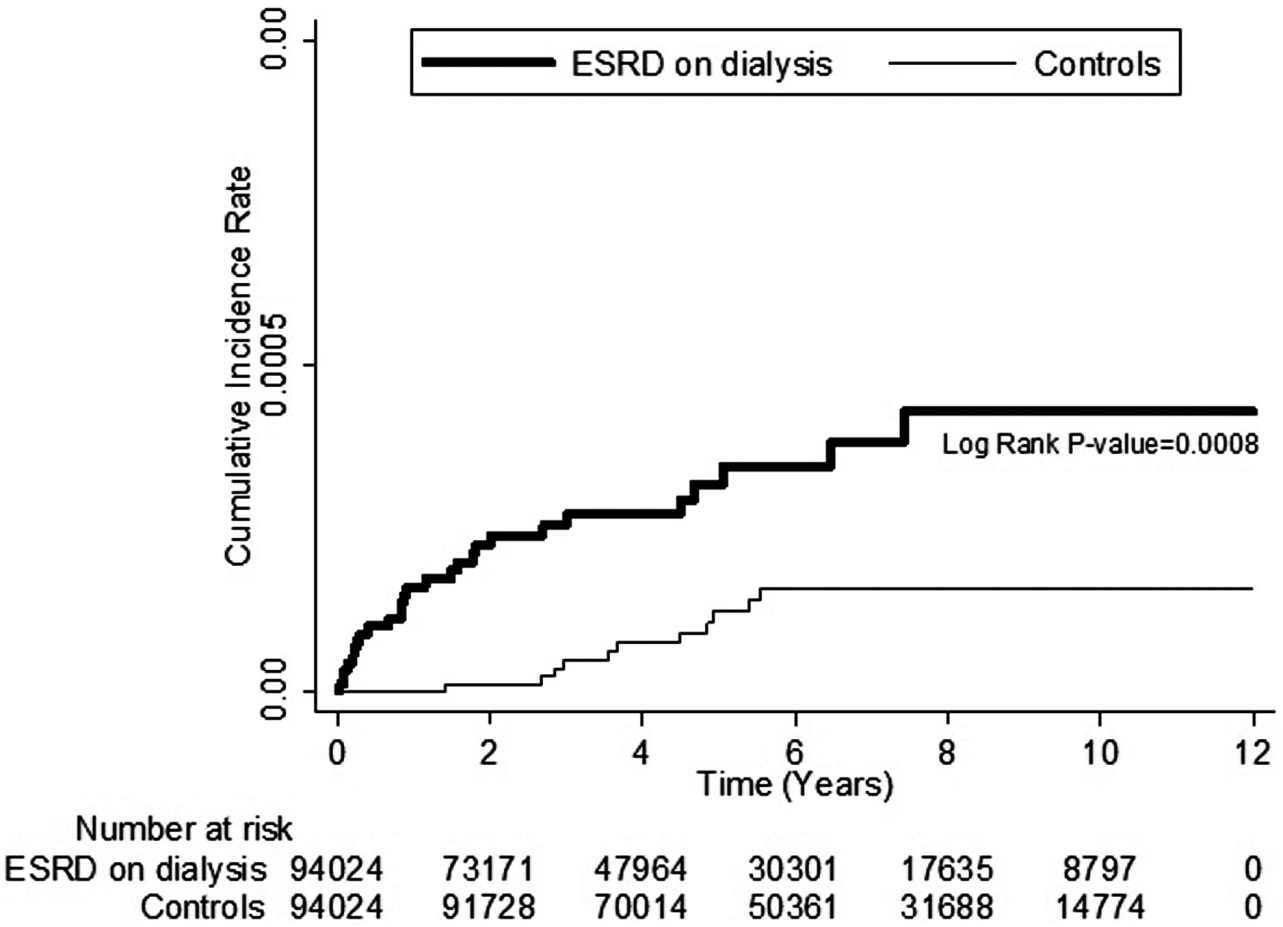
Kaplan–Meier curve of cumulative incidence of serous retina detachment in patients with end-stage renal disease (ESRD) and controls during the follow-up period.

The Kaplan–Meier survival analyses revealed higher serous retinal detachment cumulative incidence rates in the patients with ESRD on dialysis than in the control patients, and the log-rank test was also significant (P<0.001; Fig 1).

## Discussion

This cohort study is the largest-scale population-based study that has been conducted to explore the association between having ESRD while on dialysis and subsequent serous retinal detachment. We analysed 94,024 patients with ESRD on dialysis and 94,024 control subjects and found that the incidence rate of serous retinal detachment in patients with ESRD on dialysis was 3.39 times higher than in controls. Furthermore, the study also showed that the risk of serous retinal detachment for patients with ESRD on dialysis was 3.86 times higher in both groups after adjusting for diabetes mellitus, chorioretinal inflammation, and malignant neoplasm of eye.

The association between having ESRD while on dialysis and serous retinal detachment has been reported by several studies [22–26]. Goldstein presented a case report where a patient with chronic renal failure developed into bullous serous retinal detachment and informed ophthalmologists and nephrologists the possibility of bullous serous retinal detachment [26]. In one case series, Wadgi et al. reported the association of three patients with chronic renal failure patients and serous retinal detachment [25]. However, the underlying mechanism of the association between ESRD while on dialysis and serous retinal detachment has been controversial. Gass suggested that renal failure plays a more important role than dialysis in the pathophysiology of serous retinal detachment [22]. Because the fibrinous exudates were found beneath the margins of the dehiscent pigment epithelium and surrounding sub-retinal space, Gass hypothesized that large molecules, like fibrinogen, could enter the sub-retinal space. This phenomenon may be related to a focal increase in choriocapillary permeability in the uraemic state, and lead to serous retinal detachment [22]. It is worth noting that serous retinal detachment is indeed also seen after acute renal failure in patients with systemic lupus erythematosus patients not receiving haemodialysis [27]. In contrast, Troiano et al. suggested that dialysis causes variations in osmolarity and induces fluid shifts between various compartments that may have been related to altered permeability. When the fluid shifts beneath the pigment epithelium or sub-retinal space surrounding the dehiscent pigment epithelium, subsequent retinal pigment epithelial and serous retinal detachment develops [23]. In fact, intermittent haemodialysis-induced hypotension and haemodynamic intolerance has been reported in critically ill patients [28].

Our study is the largest nationwide population-based cohort study to investigate the risk of serous retinal detachment in patients with ESRD on dialysis in Taiwan. Our findings demonstrate an association between serous retinal detachment and patients with ESRD on dialysis. This finding may be associated with an alteration in choriocapillary permeability due to osmolarity variations and fluid shifts induced by dialysis. Furthermore, we found that the patients with ESRD on PD had an increased risk of serous retinal detachment than patients with ESRD on HD. We have two suggestions to explain this phenomenon. First, chronic inflammation is more common in patients with ESRD on PD than patients on HD and considered a common pathogenic mechanism for serous retinal detachment [13]. The chronic inflammation in PD patients is related to the peritoneal glucose load which leads to fluid accumulation due to cumulated peritoneal membrane damage and increased peritoneal membrane permeability [29,30]. Second, vascular injuries such as increased endothelium permeability and atherosclerotic change are more common in patients with ESRD on PD and possible predictors of the development of serous detachment [31]. The vascular impairment in patients receiving PD is associated with the peritoneal glucose load, which contributes to the formation and accumulation of advanced glycation end products, and consequent effect on extracellular and intracellular structures and function [31].

Serous retinal detachment is a common and vision-threatening retinal disorder characterised by the separation of the neurosensory retina from the pigment epithelium. In this cohort study, we evaluated diabetes, chorioretinal inflammation, and malignant neoplasm of eye in patients with ESRD on dialysis and controls and found that for both groups, diabetes was not a significant risk factor (Table 3). It should be noted that we could not evaluate the relationship between chorioretinal inflammation or malignant neoplasm of eye and serous retinal detachment because no serous retinal detachment occurred in the patients with the former conditions (Tables 2 and 3).

The elevated risk of serous retinal detachment in patients with ESRD on dialysis is an important interdisciplinary issue and close collaboration between nephrologists and ophthalmologists is important. Nephrologists should be aware of the potential visual impairment in patients with ESRD on dialysis and promptly refer potentially affected patients to ophthalmologists. The most important concerns for ophthalmologists are to know the association between serous retinal detachment and having ESRD while on dialysis; to include the various causes other than ESRD while on dialysis such as systemic inflammatory disease, infectious disease, collagen vascular disease, disseminated intravascular coagulopathy, or malignant disease in the differential diagnosis; and to give appropriate treatment according to the cause of serous retinal detachment. Once the diagnosis of dialysis-related serous retinal detachment is confirmed by an ophthalmologist, it is of utmost importance for nephrologists that effects of dialysis such as osmolarity variations or peritoneal glucose load are noted. Close cooperation between nephrologists and ophthalmologists is important to reduce the possibility of further visual impairment in serous retinal detachment in patients with ESRD on dialysis.

Our study has several strengths. The study has high statistical power and increased precision in risk appraisal because it is based on a nationwide population-based dataset, including a large sample of patients with ESRD on dialysis. Several previous studies using the same database have already been published [9,32,33]. Furthermore, our study is a cohort study analysing the incidence of serous retinal detachment in patients with ESRD on dialysis, with comparisons between cohorts over a 10-year period. Furthermore, our study has a reduced selection bias of referral centres and chance of misdiagnosis because patients with visual disturbances visit an ophthalmologist rather than a general practitioner in Taiwan. Finally, because diabetes, chorioretinal inflammation, and malignant neoplasm of eye were taken into account as confounding factors to adjust the hazard ratio of serous retinal detachment in patients with ESRD on dialysis, our results are reliable.

There are some limitations to our study. Because the sampled patients’ medical history can only be traced back to the year 1996, we cannot confirm that the controls had no history of ESRD on dialysis before January 1996. Additionally, the diagnoses of ESRD on dialysis, serous retinal detachment, and other comorbidity disorders relied on ICD-9 codes, which may lead to disease misclassification. We were also unable to account for some particular factors, including the state of hydration of these patients, and potentially higher anticoagulant or other drug usage; these factors might favour the occurrence of serous retinal detachment. Finally, the evaluation of many comorbidities associated with serous retinal detachment, including diabetes, chorioretinal inflammation, and malignant neoplasm of eye, in patients with ESRD on dialysis and controls showed that the absence of these comorbidities in the control group compromised the significant incidence ratios of these comorbidities between patients on ESRD on dialysis and control patients.

In summary, our study showed that after adjusting for comorbid diabetes, chorioretinal inflammation, and malignant neoplasm of eye, patients with ESRD on dialysis patients, especially patients receiving PD, showed a significantly higher risk of developing serous retinal detachment during the follow-up period. The linkage between serous retinal detachment and having ESRD while on dialysis is may be based on alterations in choriocapillary permeability associated with osmolarity variations and fluid shifts due to dialysis. Additionally, the higher risk of serous retinal detachment in patients with ESRD receiving PD than those receiving HD might be explained with the fact that chronic inflammation and vascular injuries are more common in PD patients. We recommend that ophthalmologists should be alert to the association between serous retinal detachment and having ESRD while on dialysis and rule out other differential diagnosis of serous retinal detachment including systemic inflammatory diseases, infectious diseases, collagen vascular diseases, disseminated intravascular coagulopathy, and malignant disease. Nephrologists should be aware of the link between the osmolarity variations in dialysis patients and increased incidence of chronic inflammation or vascular injuries secondary to improper peritoneal glucose load in PD patients, and serous retinal detachment. Close cooperation between nephrologists and ophthalmologists is important in serous retinal detachment in patients with ESRD on dialysis to reduce the subsequent visual impairment.
